# Prostate Cancer: Pathophysiology, Pathology and Therapy

**DOI:** 10.3390/cancers15010281

**Published:** 2022-12-31

**Authors:** Vasiliki Tzelepi

**Affiliations:** 1 Department of Pathology, University of Patras, 26504 Patras, Greece; btzelepi@upatras.gr; 2 Department of Histopathology and Cytology, University Hospital of Patras, 26504 Patras, Greece

Prostate cancer (PCa) is a major health care challenge in the developed world, being the most common type of cancer in men in the USA [1] and most European countries [2] and the second most common worldwide [3]. PCa shows remarkable heterogeneity in its clinical course. Some patients have indolent cancer that will never progress, whereas others have a remarkably aggressive disease with rapid progression to metastases and resistance to therapy [4,5], making PCa the fifth most common cause of cancer-related death worldwide [3]. In between are patients with an initially localized disease that will progress to a metastatic, incurable disease after a variable time period [4]. In this Special Issue, recent advances in precision medicine approaches for PCa in regard to clinical, pathologic, molecular and therapeutic parameters are presented. 

In a review paper, Cimadarone et al. [6] summarize the novel prognostic and predictive tissue-based biomarkers for PCa developed in 2021. They discuss the contemporary PCa grading, the advantages offered by computational pathology and artificial intelligence, the morphologic and immunohistochemical features of aggressive variants of PCa, and the molecular markers that can be used in the clinic to predict an aggressive disease or a response to specific therapies. 

The clinical heterogeneity of prostate cancer is a reflection of its molecular heterogeneity and the emergence of lineage plasticity, which is the ability of neoplastic cells to switch between distinct lineages and phenotypic cell states by adapting to their environment [7]. The epithelial-to-mesenchymal transition is an example of lineage plasticity and represents an important mechanism of tumor progression and therapy resistance. In a review paper in this issue, Papanikolaou et al. [8] discuss the molecular pathology of the epithelial-to-mesenchymal transition in PCa, the pathways involved in its emergence, and its effect on PCa aggressiveness and therapy resistance, as well as potential opportunities for therapeutic targeting.

Currently, prostate-specific antigen (PSA) levels, clinical stages and the biopsy Gleason Score are the main parameters used to stratify patients into risk categories at initial presentation, albeit with modest specificity and sensitivity, and are used to decide on the appropriate therapy [9]. The Gleason Score, originally described by Donald Gleason in 1974 [10], has been continuously refined over the years [11,12] in an effort to enhance its prognostic value. Reproducibility in PCa grading by pathologists is of paramount importance for accurate risk classification and therapy selection. However, in a nationwide survey among 41 laboratories and >38,000 needle biopsy reports, which was completed by Flach et al. and presented in this Special Issue [13], a significant inter- and intra-laboratory variation in the daily practice of PCa grading was noted, irrespective of the PCa case volume diagnosed by the pathologist and corrected by PSA levels atdiagnosis, year of diagnosis, age, number of biopsies taken, and number of positive biopsies. These findings highlight the need for better standardization of PCa grading, for example, through training and the use of artificial intelligence.

Apart from the Gleason Score, specific morphologic patterns of PCa, i.e., cribriform and intraductal carcinoma (IDC), as well as tertiary patterns, seem to have additional prognostic significance. However, confusing guidelines exist in regard to how this information is conveyed to clinicians [14,15,16]. In a paper in this Special Issue [17], Tzelepi et al. show that, depending on the grading criteria used (handling of IDC and tertiary patterns), the final grade would be different in a number of cases, with a three-point difference in a minority of them. This may be a source of confusion amongst both pathologists and urologists. They also provide support for IDC incorporation into the final grade, even though they recognize the need for additional studies with survival as the end point. 

Radiotherapy (RT) is a therapeutic option for localized prostate cancer, with fractionation and dose depending on the clinical scenario [9]. Previous studies have shown that external beam radiotherapy with moderate hypofractionation is effective in low-risk, intermediate-risk and high-risk localized prostate cancer. In their study, di Muzio et al. [18] present their experience with pelvic lymph nodal irradiation through a moderately hypofractionated, high-dose, simultaneously integrated boost to the prostate and seminal vesicles and long-term androgen-deprivation therapy in unfavorable intermediate-, high- and very high-risk localized prostate cancer patients. They confirmed the good clinical outcomes and acceptable toxicity of this approach, in terms of ten-year biochemical-relapse-free survival and disease-free survival, with a longer follow-up (median of 107.6 months (IQR: 78.35; 136.10)) than that used in previous studies, and they identified a Gleason Score ≥8 as an independent predictor of biochemical relapse.

With conventional RT approaches, a prescribed dose is delivered to the prostate. With biologically targeted approaches, the specific characteristics of the tumor are considered in order to maximize tumor control while limiting the toxicity in adjacent normal tissues. The inability to spatially map tumor characteristics has limited the wide application of this approach. In their study, Hen et al. [19] compare in silico radiotherapy with a tumor and hypoxic sub-volume boost by using mpMRI-derived cell density and hypoxia maps, to conventional approaches. Their results showed that biological optimization improved rectal and bladder sparing, supporting further investigations of biologically targeted approaches by taking advantage of the knowledge on the spatial distribution of tumor heterogeneity.

Androgen deprivation therapy (ADT) using LHRH agonists/antagonists that cause pharmacologic castration, with or without androgen receptor (AR) antagonists, represents the main course of therapy for patients that present with metastatic disease and for patients that develop biochemical recurrence after their initial therapy [20]. ADT is not without side effects, with cardiovascular events, including myocardial infarction and cardiovascular-related mortality, being the most important [21]. Dementia has also been associated with ADT, albeit the reports are controversial. In this issue, Liu et al. [22] describe a population-based cohort study of 129,126 men with PCa, with data obtained from the National Health Insurance Database of Taiwan and The Health Improvement Network Database of the United Kingdom, and show that there is no difference in the incidence rates of dementia between patients receiving ADT and those that are ADT naïve, nor is there a difference in any cumulative dose effect between ADT and dementia.

The majority of patients will respond well to androgen deprivation treatment, but the tumor will, eventually, recur, leading to castrate-resistant prostate cancer (CRPC) [23]. Second generation antiandrogens and various other therapies are available for these patients, which result in a significant prolongation of patients’ survival [20]. Predictive markers as to the best sequential order of these therapies are largely lacking. In their study, Hsieh et al. [24] provide evidence that metastatic CRPC (mCRPC) patients receiving enzalutamide with a high tumor burden, defined as either appendicular bony or visceral metastasis, showed fewer good PSA response rates, fewer rates of partial radiological response and stable disease, and a shorter progression-free survival duration compared to low-tumor-burden patients. Although further studies are needed to identify a model that will inform therapeutic decisions, this study, as well as others [25,26], highlight tumor burden as a clinical factor to be considered when deciding upon an optimal therapeutic strategy. 

Similarly, in an effort to identify markers of disease aggressiveness to guide therapeutic decisions, Delanoy et al. [27] present a post hoc analysis of PROSELICA, a large phase III randomized study (NCT01308580) that evaluated two doses of cabazitaxel (the standard dose of 25 mg/m^2^ every 3 weeks and a lower dose of 20 mg/m^2^ every 3 weeks) in mCRPC patients previously treated with docetaxel [28]. The authors assessed the prognostic value of the type of disease progression at cabazitaxel initiation in a post-docetaxel setting and confirmed that pain progression at cabazitaxel initiation was associated with clinical and biological features of aggressive disease and worse outcomes, andwas a better predictor of disease aggressiveness than PSA progression.

CRPC emergence involves the development of AR mutations, with the majority being located at the ligand-binding domain of the molecule [29], and the expression of AR splice variants, most being constitutively active despite lacking the ligand-binding domain [30]. Both mechanisms render most first- and second-generation AR-targeting approaches (that directly or indirectly target the ligand-binding domain) ineffective. Darolutamide, a second-generation hormone therapy, is an AR antagonist that is structurally distinct compared to other AR antagonists, including enzalutabite and apalutamide, and has been proposed to inhibit AR mutant proteins that are resistant to other antagonists. In their paper, Lallous et al. [31] examine the effect of darolutamide, as well as the commonly used antiandrogens bicalutamide and enzalutamide and the major endogenous steroids DHT, estradiol, progesterone and hydrocortisone, on 44 AR mutants (including data presented in their previous work [32]) and identified only one mutant that was not inhibited by darolutamide, highlighting this compound as a potential therapeutic option for CRPC patients with AR mutations.

Since the ligand-binding domain of AR is the most affected under the pressure of therapy and since most of the AR transcriptional activity is mediated via its N-terminal domain (NTD), targeting the NTD domain represents a promising therapeutic strategy for CRPC patients. In their paper, Ban et al. [33] characterize novel AR NTD-targeting molecules. Based on a molecule identified in their previous work, VPC-2055, they synthesized and tested 110 novel compounds. Among them, eight were found to be active in physiologic concentrations and one, VPC-220010, was three and five times more potent than VPC-2055 and EPI-001, a known NTD inhibitor, respectively, and was further extensively characterized in regard to the inhibition of AR activation and PCa cell-line viability, providing preclinical evidence for its potential clinical utility.

When progressing, PCa largely metastasizes to the bones, and cancer cell osteomimicry has been implicated in this process. MINDIN, an extracellular matrix protein, has been shown to induce osteomimicry and prostate cancer progression [34]; however, its mechanisms of action have not been elucidated. In their paper, Alvarez-Carrion et al. [35] provide evidence that MINDIN downregulates the expression of NHERF-1, a scaffold protein that interacts with various proteins including receptors, transporters and cytoplasmic signaling proteins, thereby enhancing a variety of cellular processes including cell proliferation and migration.

Another mechanism by which AR is activated in the CRPC setting is via crosstalk with growth factor receptor pathways, including the PI3K/AKT pathway, through PTEN deletion [36]. Apalutamide is a second-generation antiandrogen that binds to the ligand-binding domain of AR and inhibits its nuclear translocation. It their paper, De Velasco et al. [37] characterize the antitumor activity of apalutamide in genetically engineered, *Pten*-deficient PCa mouse models. They showed that apalutamide demonstrated antitumor activity in models of castration-naïve PCa (*Pten*-deficient) and CRPC (*Pten/Trp53* double knockout). Upregulated aberrant AR and a phosphorylated S6 and proline-rich Akt substrate of 40 kDa (PRAS40) were seen in the surviving cancer cells in CRPC. Strong synergy was observed between the pan-AKT inhibitor GSK690693 and apalutamide in vivo in castration-naïve PCa, but not in CRPC. Further studies of apalutamide combinations with AKT and the inhibition of other pathways are needed.

AR-independent mechanisms have also been implicated in CRPC development. In addition, a transformation from adenocarcinoma, the prototype of PCa, to neuroendocrine carcinoma (NECa), an aggressive tumor type characterized by lytic bone metastasis, splanchnic metastasis and low PSA for disease burden, is seen in some, but not all, of the CRPC cases under the pressure of therapy [38]. Elucidating the mechanisms of AR-independent progression is of paramount importance for the discovery of therapeutic targets for this devastating type of the disease. In this Special Issue, Xu et al. [39] show that the expression of ELOVL5, the key enzyme of long-chain polyunsaturated fatty acid elongation, was elevated in NECa cell lines, and its knock down diminished their neuroendocrine phenotype and enzalutamide resistance, whereas its overexpression enhanced enzalutamide resistance in PCa cell lines in vitro and in vivo. These findings highlight ELOVL5 as a potential therapeutic target for PCa patients with the NECa phenotype.

This Special Issue includes some important developments in PCa research for the implementation of personalized medicine in the treatment of PCa patients and for using clinical, morphologic and molecular markers to inform therapeutic decisions. 
